# Prior cardiac events and the interpretation of ECG-based artificial intelligence for inducible myocardial ischaemia

**DOI:** 10.1093/ehjdh/ztag077

**Published:** 2026-05-18

**Authors:** Liam Munir, Adele Cai

**Affiliations:** School of Medicine and Dentistry, Griffith University, 1 Parklands Drive, Southport, 4215 Gold Coast, Australia; School of Medicine and Dentistry, Griffith University, 1 Parklands Drive, Southport, 4215 Gold Coast, Australia

Dear Dr Nico Bruining,

We read with interest the study by Lim and colleagues,^1^ in which a convolutional neural network trained on resting 12-lead electrocardiograms (ECGs) identified patients with inducible myocardial ischaemia with an area under the receiver-operating characteristic curve (AUROC) of 0.90 in internal testing, with comparable performance across multi-institutional validation cohorts. The work is methodologically careful and clinically ambitious, particularly in its framing as a potential screening tool that might extend to asymptomatic individuals. We wish to draw attention to a specific feature of the case definition that, in our view, materially affects how the reported performance should be interpreted against this stated use case.

The ‘ischaemia group’ was defined as patients who underwent revascularization (percutaneous coronary intervention [PCI] or coronary artery bypass grafting [CABG]) for silent ischaemia, stable angina, or unstable angina. As reported in the authors’ *Table 2*, the test-set ischaemia group included 21 patients with a previous myocardial infarction, 108 with prior PCI, and 22 with prior CABG—together approximately one-quarter of test cases. By contrast, the no-ischaemia group explicitly excluded any history of myocardial infarction or revascularization (*Figure 1* of Lim *et al.*). This design asymmetry is important because prior myocardial injury and intervention leave well-characterized, persistent ECG signatures such as pathological Q waves, fragmented QRS, conduction delays after cardiac surgery, and T-wave memory after pacing or PCI. Such features are readily detectable by cardiologists and even more reliably by a deep neural network.^2,3^ Detecting the ECG signature of past myocardial injury is a distinct task from detecting the subtle resting markers of latent inducibility in structurally preserved myocardium, which is the biological premise of the proposed screening application.^4^

The subgroup sensitivities reported in *Table 2* of Lim *et al.* are consistent with a substantive contribution of this prior-event signal to the headline performance (*Table 1*). Sensitivity was highest in patients with prior myocardial infarction (95.2%) and prior CABG (90.9%), and was lowest precisely in the subgroup that most closely approximates the intended asymptomatic-screening target: Silent ischaemia (78.5%). The unmatched model (AUROC 0.90) also outperformed the age- and sex-matched model (AUROC 0.85); the authors appropriately note that this may reflect clinically relevant age-related risk information, but it is equally consistent with residual demographic and comorbidity shortcuts that matching on age and sex alone cannot eliminate.^5,6^ AI-ECG models have previously been shown to recognize prior myocardial infarction with high accuracy,^7^ confirming that this is a learnable signal that could dominate the model output if permitted to enter the training label.

**Table 1:** Model sensitivity within the test-set ischaemia group (*n* = 604) stratified by clinical diagnosis and prior cardiac history (data extracted from *Table 2* of Lim *et al.*). Sensitivity is highest in patients with prior revascularization or infarction, and is lowest within the silent-ischaemia subgroup that most closely approximates the intended asymptomatic-screening population. CI, confidence interval

Subgroup	*n*	Sensitivity (95% CI)
**By clinical diagnosis**		
Silent ischaemia	79	78.5% (67.8–86.9)
Stable angina	345	86.1% (82.0–89.6)
Unstable angina	180	81.7% (75.2–87.0)
**By prior cardiac event**		
Previous myocardial infarction	21	95.2% (76.2–99.9)
Previous percutaneous coronary intervention	108	83.3% (74.9–89.8)
Previous coronary artery bypass grafting	22	90.9% (70.8–98.9)

This is not a generic concern about selection bias but a specific, testable consequence of how cases were assembled, and one that is readily addressable within the existing dataset without a new prospective study. A re-analysis restricted to patients without a history of myocardial infarction or prior revascularization, and ideally further restricted to ECGs obtained before any unstable presentation, would allow the reported performance to be attributed to inducible ischaemia specifically, rather than to the ECG echoes of previous events. If the AUROC is substantially preserved in such a re-analysis, the screening claim is materially strengthened. If it is not, the findings would still represent a valuable advance in the recognition of established ischaemic heart disease, but would not support application in asymptomatic screening populations in whom the prevalence of prior events is by definition negligible.^8^

A related consideration concerns the comparator group, which was drawn from patients with both 0% epicardial stenosis and a coronary artery calcium score ≤100 on coronary computed tomography angiography, and whose mean age was approximately eleven years younger than that of the ischaemia group. Although the age- and sex-matched sensitivity analysis is reassuring at an AUROC of 0.85, matching addresses only a subset of the dimensions along which these cohorts differ, not cardiovascular risk factor burden, medication exposure (e.g. β-blockers or antihypertensives that influence the ECG), or left ventricular hypertrophy from long-standing hypertension. Reporting the performance obtained after also adjusting for these covariates, or by stratified sensitivity analyses, would further strengthen the interpretation.

We offer these observations in the recognition that pragmatic case definitions are often unavoidable in AI diagnostic studies, and that Lim *et al.* have assembled an impressive, multi-centre dataset. However, the clinical claim that is most likely to influence practice (screening of asymptomatic individuals) requires evaluation in a case spectrum considerably narrower than the one used here. Clarifying model performance within that spectrum, using data already in hand, would provide a more informative foundation for the prospective screening trials that the authors and we both look forward to.
